# Cardioprotective effect of Yiqi Huoxue decoction on post-myocardial infarction injury mediated by Ca^2+^ flux through MAMs

**DOI:** 10.3389/fcvm.2025.1596757

**Published:** 2025-07-29

**Authors:** Yufei Li, Tianhui Du, Yunshu Zhang, Yang Lu, Xinyi Li, Weibin Xie, Shuwen Guo

**Affiliations:** ^1^ School of Traditional Chinese Medicine, Beijing University of Chinese Medicine, Beijing, China; ^2^ Department of Cardiovascular Diseases, Fangshan Traditional Medical Hospital of Beijing, Beijing, China

**Keywords:** Yiqi Huoxue decoction (YQHX), cardiomyocytes, myocardial infarction (MI), MAMS, IP3Rs-GRP75-VDAC1 complex

## Abstract

**Background:**

Mitochondria-associated membranes (MAMs) regulate cellular Ca^2+^ and contribute to cardiovascular disease pathogenesis. The IP3R-GRP75-VDAC1 complex is the primary MAMs pathway regulating Ca^2+^ flux and cardiomyocyte calcium homeostasis. Yiqi Huoxue decoction (YQHX), a Traditional Chinese Medicine formula, shows potential for myocardial infarction (MI) prevention and treatment. However, YQHX's regulation of MAMs and associated Ca^2+^ mechanisms in MI remains unclear.

**Methods:**

MI rat and oxygen-glucose deprivation cardiomyocytes model were used to mimic myocardial ischemia in human. *in vivo*, Rats were randomly divided into Sham, Model, YQHX (8.2 g/kg) and Perindopril (10 mg/kg) groups. 28 days after MI, echocardiography, HE, Masson staining and transmission electron microscopy detections were performed to observe cardiac functions and morphology. The effects of YQHX on H9c2 cell viability, mPTP and Ca^2+^ levels were examined *in vitro*. Proteins located at MAMs including Cyclophilin D (CypD), Mitochondrial Calcium Uniporter (MCU), Sigma-1 Receptor (Sig-1R), and Neurite Outgrowth Inhibitor B (NOGO-B) are abundantly expressed in myocardial tissue. Consequently, these proteins, along with components of the IP3Rs-GRP75-VDAC1 complex, were detected using WB and qPCR. Mitofusin 2 (Mfn2), which regulates mitochondrial function and Ca^2+^ flux and is widely expressed at MAMs, was assessed using immunofluorescence.MKT-077, an agent known to disrupt the IP3Rs-GRP75-VDAC1 complex, was employed to investigate the mechanism of YQHX on the complex.

**Results:**

YQHX improved cardiac function and attenuated pathological changes *in vivo*. It ameliorated MAMs ultrastructure and function, enhancing CypD, MCU, Sig-1R, and NOGO-B expression while reducing IP3R2, GRP75, and VDAC1. *in vitro*, YQHX significantly increased viability, reduced oxygen-glucose deprivation-induced mPTP opening and Ca^2+^ levels, upregulated CypD, MCU, Sig-1R, and NOGO-B, and downregulated IP3R2, GRP75, and VDAC1. YQHX also restored MAMs morphology, decreased mPTP opening and Ca^2+^ levels, and reversed GRP75 downregulation blocked by MKT-077 under oxygen-glucose deprivation.

**Conclusions:**

YQHX exerts cardioprotection against hypoxia by regulating Ca^2+^ homeostasis and preserving MAMs structure, function, and associated protein expression.

## Introduction

Cardiovascular disease (CVD) is the leading cause of death worldwide. The prevalence and case fatality rate for CVD are particularly high (1) CVD has become the first cause of mortality among Chinese urban and rural residents, with 46.66% in urban and 43.81% in rural area (2), causing serious social and economic burdens. Through increasing urbanization and economy, the lifestyle of people in China has changed profoundly. Unhealthy lifestyle habits such as physical inactivity, unhealthy diet, and smoking caused the rising incidence of CVD showing a trend of younger age due to the high incidence of dyslipidemia, obesity, hypertension, and diabetes (3).

Among various cardiovascular diseases, the incidence of coronary artery disease is approximately 3% (4) and 7% to 10% of acute myocardial infarction occurs in young individuals under 45 years of age (5). Acute myocardial infarction (AMI) is a severe type of CVD, its mortality rate in China has been increasing with 120.18/100,000 and 128.24/100,000 in urban and rural areas, respectively (6). The COVID-19 pandemic in 2019 led to a higher mortality rate, increased risk of myocardial infarction (MI), and the occurrence of cardiovascular complications such as decreased left ventricular function and arrhythmia in patients with heart injury due to the infection (7). Therefore, new therapeutic targets of MI must be sought.

Cardiomyocyte contraction-relaxation cycles are regulated by intracellular Ca^2+^ levels, calcium balance of organelles ensures the normal structure and function of cells (8). Endoplasmic reticulum (ER) is a major intracellular Ca^2+^ store, and mitochondria are essential organelles that buffer cytoplasmic calcium. Mitochondria-associated membranes (MAMs), as physical and functional interfaces, serve as hotspots for Ca^2+^ transfer between the ER and mitochondria (9). MAMs plays a crucial role in cardiovascular diseases such as MI, ischemia reperfusion, and heart failure by participating in processes like calcium homeostasis regulation, endoplasmic reticulum stress, and mitochondrial dynamics. They are currently one of the research hotspots (10, 11). An imbalance in Ca^2+^ homeostasis is a vital cause of the cardiovascular disease, the regulation of Ca^2+^ on MAMs is a critical treatment target (12, 13). IP3Rs-GRP75-VDAC1 complex, exerts an important function of calcium homeostasis in myocytes, is the main pathway of Ca^2+^ flux in MAMs (14). Among the proteins of complex, inositol-1,4,5-trisphosphate receptors (IP3Rs) is a ligand-gated calcium channel that is primarily localized to the endoplasmic/sarcoplasmic reticulum (ER/SR). As a family of calcium channels, IP3Rs are ubiquitously expressed in all tissues. In the heart, IP3Rs have been associated with regulation of cardiomyocyte function in response to a variety of neurohormonal agonists, including those implicated in cardiac disease (15). Voltage-dependent anion channel 1 (VDAC1) is a multi-functional channel, which mediates metabolites, nucleotides, and Ca^2+^ transport, controlling energy production and ER-mitochondria crosstalk. VDAC1 is responsible for the passage of Ca^2+^ to and from the intermembrane space to facilitate Ca^2+^ signaling and contributing to ER-mitochondria contacts, where Ca^2+^ released by IP3 activation of IP3Rs in the ER is directly transferred to MAMs via VDAC1 (16). Glucose-related protein 75 (GRP75) is a heat shock protein that regulates a wide range of cellular processes, including cell survival, growth, and metabolism (17). As a crucial mitochondrial protein, around 30% of GRP75 in the cell is located in other cellular compartments such as the cytoplasm, ER that connects the calcium channels on the surface of between ER and mitochondria (18, 19). Thus, further investigation is warranted to determine whether MAMs and IP3R2-GRP75-VDAC1 complex in myocardial cells could serve as novel clinical targets for pharmacological intervention in myocardial infarction, by modulating intracellular calcium homeostasis after AMI.

From the perspective of TCM, MI is named as “chest pain or cardialgia” and its general pathogenesis is Qi deficiency and blood stasis. Yiqi Huoxue decoction (YQHX), consists of Angelica sinensis, ginseng, Astragalus membranaceus, Ligusticum chuanxiong, and Panax notoginseng is created based on the TCM principle of invigorating qi and activating blood (20). In a similar study by YaoPing et al. revealed that YQHX improved calcium transport of cardiomyocyte in HF rats and ameliorated cardiomyocyte apoptosis by suppressing ER stress (21). YQHX also improved Ca^2+^ transport ability of cardiomyocyte and diastolic function, prevented ventricular arrhythmogenesis (22).

Our previous research has confirmed that 20 compounds in YQHX may be the main active ingredients for the treatment of ischemic heart disease. NHE1, Ca MKII and PKC are the parts of the 20 compounds (23). We have also confirmed that the impact of YQHX on SR calcium leakage and mitochondrial calcium-regulating proteins after MI is consistent with the susceptibility to arrhythmias and the manifestation of improved cardiac function (24).

Therefore, based on previous research, this study aims to explore the impact of YQHX on Ca^2+^ homeostasis through MAMs and to discover additional therapeutic targets for the treatment of MI.

## Methods

### YQHX preparation

YQHX (Astragali 50 g, Ginseng 10 g, Angelicae Sinensis 15 g, Chuanxiong Rhizoma 5 g and Notoginseng 6 g) used in animal experiments were purchased from Dongzhimen Hospital of Beijing University of Chinese Medicine. According to the preparation process of oral Chinese medicine decoction, a continuous reflux extraction was performed for 1 h using 10 times the amount of boiling distilled water (g/m), repeated three times. The aqueous extract was filtered and concentrated to a final volume of 100 ml, such that each milliliter of the medicine corresponds to 0.82 g of crude drug, and then administered to rat through gastric lavage at a dose of 8.2 g/kg/d, which corresponds to a daily dose for adults (20, 25–27). YQHX decoction for cell experiments was prepared by lyophilizing the supernatant from animal experiments into a powder. The powder was vortex-mixed and completely dissolved in a 37°C water bath, followed by filtration through a 0.22-μm membrane filter (Millipore, USA). The filtrate was sealed and stored at 4°C until use.

HPLC-LTQ Orbitrap MS and DDAMS2 data acquisition methods were employed to analyze the components of the aqueous extract of YQHX. 87 compounds have been identified, primarily consisting of triterpenoid saponins and flavonoids. Ginsenosides, anthracene glycosides, astragalosides, wugui glycosides, and panaxosides are included (26).

### Animal experiment

Adult male Sprague Dawley (SD) rats of SPF grade (200 ± 20 g) were obtained from Beijing Vital River Laboratory Animal Technology Co., Ltd. (license number: SCXK2021-0011). Rats were acclimatized for 5 days in the barrier facility of the Animal Experiment Center at Beijing University of Chinese Medicine prior to MI surgery. Environmental conditions were maintained as follows: daily temperature fluctuation ≤4℃ (range: 20–26℃), relative humidity 40%–70%. Left anterior descending coronary artery (LAD) ligation surgery was performed for MI model rats as described previously (27–29). First, the rats were anesthetized with an intraperitoneal injection of 1% pentobarbital sodium at a dose of 40 mg/kg and shaved chest for surgical preparation. Then, rats were connected to ventilator after tracheal intubation (tidal volume of 0.6 ml, a respiratory ratio of 1:2, and a respiratory rate of 85 breaths per minute). Thoracotomy was then performed between the 3rd and 4th intercostal spaces, and LAD was ligated. Except the LAD ligation, the operation was the same procedure in Sham group. Finally, electrocardiographic (ECG) monitoring was conducted 24 h postoperatively. The rats were randomly divided into sham group (S, *N* = 9), and infarct group. Rats with ST segment elevation in the limb and more than 4 Q-wave numbers were confirmed the successful replication of MI model. The rats with the same Q-wave number were randomly assigned to 3 group: myocardial infarction model (M, *N* = 9), myocardial infarction model + YQHX (Y, *N* = 9), and myocardial infarction model + perindopril (P, *N* = 9). Administration dosage of YQHX was 8.2 g/kg/day while perindopril (Servier Co., Ltd., Tianjin, China) was administered 0.4 mg/kg/day via gavage at 9:00 am on the second day after the MI modeling, and the administration lasts for 7 and 28 days respectively as previously described (27).

### Cell culture and treatment

H9c2 cells (Servicebio, Wuhan, China) are cultured in the H9c2 special medium (Servicebio, Wuhan, China) in a CO_2_ incubator (Thermo, Scientific, Bremen, Germany). Cells were replaced with low-sugar serum-free DMEM (Invitrogen, California, USA) at the confluence of 80% and placed in a three-gas box (Thermo, Scientific, Bremen, Germany) for 24 h of culture (95% N_2_, 5% CO_2_, and O_2_ concentration ≤1%) to prepare a model of glucose and oxygen deprivation cells. After the various concentrations of YQHX (ranging from 50 μg/mL to 1,000 μg/mL) CCK-8 assay and cell damage test, the experiment was divided into 5 groups: the control group (C), the model group (M), and Y10, Y5, Y2 groups. YQHX was diluted to concentrations of 1,000 μg/mL, 500 μg/mL, and 250 μg/mL for Y10, Y5, Y2 groups respectively. A mixture of MKT-077 (MedChemExpress, New Jersey, America) and Y5, which is medium concentration of YQHX was used for the complex research.

### Echocardiography

Echocardiographic exams were conducted at day 7 and day 28 after MI modeling (Visual Sonics Inc., Toronto, Canada). The rats were anesthetized with intraperitoneal injection of 1% sodium pentobarbital, and the chest skin was prepared in the supine position. An average of three consecutive cardiac cycles were captured for each rat. The following parameters were measured: left ventricular short axis rate (FS), left ventricular ejection fraction (EF), left ventricular end-systolic volume (LVESV), left ventricular end-diastolic volume (LVEDV) and left ventricular end-systolic diameter (LVDs), left ventricular end-diastolic diameter (LVDd).

### Transmission electron microscopy

After perfusion of the rat heart with 0.9%NaCl (Servicebio, Wuhan, China, G4702–500ML) and 4%paraformaldehyde (Coolaber, Beijing, China, SL331828400), tissues in the infarct border zone were excised into less than 1 mm^3^ cube and fixed in a pre-cooled 2.5% glutaraldehyde (Labcom, Fuzhou, China, 20230921) solution for 2 h. Similarly, H9c2 cells were fixed with 2.5% glutaraldehyde for 1 h, then scraped off, centrifuged at 500–800 rpm for 5 min, and fixed for 1 h. After three washes, heart tissues and H9c2 cells underwent further processing. Copper grids were stained with 2% uranyl acetate, followed by embedding in epoxy resin. The embedded samples were then heated to 60℃ for polymerization. Finally, images were captured using a transmission electron microscope (Hitachi-H-7650B, Tokyo, Japan).

### Histologic examination

Rat hearts were fixed in 4% paraformaldehyde for 24 h, followed by paraffin embedding and sectioning into 5-μm-thick slices for subsequent analyses. For general histology, tissue sections were deparaffinized, subjected to hematoxylin and eosin (HE) staining, dehydrated through graded ethanol series, cleared in xylene, and coverslipped. Masson trichrome staining was performed following the manufacturer's instructions: after completing the staining protocol, sections were rinsed with distilled water, differentiated with 1% glacial acetic acid, and permanently mounted with neutral balsam for microscopic examination. HE staining serves as a fundamental technique for evaluating myocardial pathological changes, whereas Masson trichrome staining is employed to assess myocardial fibrosis by visualizing collagen deposition.

### Cell viability assay

H9c2 cells were cultured in a 96-well plate, 10% CCK-8 (Biorigin, Beijing, China) was added to assess the effects of different concentrations of YQHX and MKT-077 on cell viability. After incubating the cells with CCK8 for 2 h, the absorbance values of the samples were accurately measured at 450 nm using a microplate reader.

### Ca^2+^ detection

H9c2 cells were cultured in a 96-well plate, the Fluo-4 AM working solution, diluted at a ratio of 1:500 was added for Ca^2+^ detection (Beyotime, Shanghai, China). Briefly, the Fluo-4 AM working solution was added to the cell culture wells and incubated at 37°C for 30 min in the dark. After washing 3 times with PBS, the cells were incubated for an additional 15–30 min at 37°C under 5% CO₂. Fluorescence images were then captured using a confocal microscope.

### mPTP detection

H9c2 cells were cultured in a 96-well plate. Pre-prepared Calcein AM staining solution, fluorescence quenching working solution, and Ionomycin control solution were added separately (Beyotime, Shanghai, China). Cells were incubated at 37°C in the dark for 30 min, followed by replacement with fresh culture medium pre-warmed at 37°C to ensure sufficient hydrolysis of Calcein AM by intracellular esterases, generating green fluorescent Calcein. Then the cells were washed 3 times with PBS, and buffer was added. Images were then recorded using a fluorescence microscope. All cell experiments, including CCK-8 assay, Ca^2+^ detection, and mPTP opening assay, were performed in triplicate.

### Immunofluorescence

The rat heart was fixed, embedded in paraffin, and cut into thin slice. After cell membrane permeabilization and serum blocking, the following primary antibody, Mfn2 (Proteintech, Chicago, USA), IP3R2 (Santa Cruz, California, USA), GRP75 (Proteintech, Chicago, USA), VDAC1 (Affinity Biosciences, Inc., USA) were incubated overnight in the dark at 4°C. The secondary antibody (goat anti-rabbit IgG 1:200, goat anti-mouse IgG, 1:400, goat anti-rabbit IgG 1:400) were then incubated for 50 min at room temperature in the dark. After counterstaining the cell nuclei with DAPI using the DAPI staining solution, the slides were mounted with an anti-fade mounting medium. Images were recorded using a fluorescence microscope.

### qPCR

TRIzol reagent was used to extract total DNA used for reverse transcription from myocardial tissue. The reverse transcription system are as follows: 5× Reaction Buffer (4 μl), Oligo(dT)18 Primer (50 μM) (1 μl), RT Enzyme Mix (1 μl), RNase-free water (12 μl), and mRNA (2 μl). The reverse transcription reaction is performed in PCR instrument (25℃,300s; 42℃,1800s; and 85℃, 5s.) The sequences for the primers used are as follows: MCU: forward, 5'-CCCCTGGAGAAGGTACGGAT-3', reverse,5'-GGTGACCGGTTCCATGATGT-3', CypD: forward, 5’-CGCTTTCCTGACGAGAACTT-3', reverse,5'-ACATCCATGCCCTCTTTGAC-3', Sig-1R: forward, 5’-GCAGTGGGTGTTTGTGAACG-3', reverse,5'-GCCCAGTATCGTCCCGAATG-3', NOGO-B: forward, 5’-GAACTGAGGCGGCTTTTCTT-3', reverse,5'-TGATCTATCTGCACCTGATGCC-3', IP3R2: forward, 5’-ACCTCTGCGTGTCCAATAGC-3', reverse,5'-CATGGACACCAGCTTCGTCT-3', GRP75: forward, 5’-GACGAGGATGCCCAAGGTTC-3', reverse,5'-CAGCCAACACACCTCCTTGA-3', VDAC1: forward, 5’-AGCCTCCTCGCCGCAA-3', reverse,5'-GCACAGCCATGTTCTCGGA-3', GAPDH: forward, 5’-AGACAGCCGCATCTTCTTGT-3', reverse,5'-CTTGCCGTGGGTAGAGTCAT-3'. qPCR was performed on the Quantagene q225 PCR instrument. The qPCR denaturation involved 42 cycles (95℃, 30s; 95℃, 15s; 60℃, 30s; 75℃, 30s) GAPDH served as the internal reference in this experiment, and the relative mRNA levels were calculated using the 2-ΔΔCt method.

### Western blotting

Marginal heart tissues were washed with pre-cooled PBS and added lysis buffer to extract the proteins. Similarly, lysis buffer was added to H9c2 cells. Both the tissue and cell samples were then assayed for protein concentration using the BCA method. The protein samples were first loaded onto gels and then transferred to membranes, blocked for 2 h. The membranes were following incubated overnight at 4°C with the primary antibodies, CypD (Affinity Biosciences, Inc., USA), MCU, Sig-1R and NOGO-B (Proteintech, Chicago, USA). GAPDH and β-actin were employed as loading controls. Following the incubation, the membranes were washed with TBST and incubated with secondary antibodies (selected based on the type of primary antibody, either goat anti-rabbit or goat anti-mouse, diluted in 3% skim milk at a ratio of 1:50,000) for 30 min. Finally, the membranes were added with the ECL mixed solution and placed in a developing tray for exposure and image capture. Grayscale values of the blots were analyzed by Image J software processing system. For each protein, Western blot and qPCR experiments were performed in triplicate.

### Statistical analysis

Experimental data were analyzed using SPSS 22.0 software (IBM, Armonk, USA). Measurement data were presented as mean ± SD. Student's *t*-test, one-way ANOVA, or repeated-measures ANOVA were employed to compare values between groups. A statistically significant difference was determined when the *P*-value was less than 0.05.

## Results

### YQHX improved cardiac structure and dysfunction after MI

**Figure 1:**
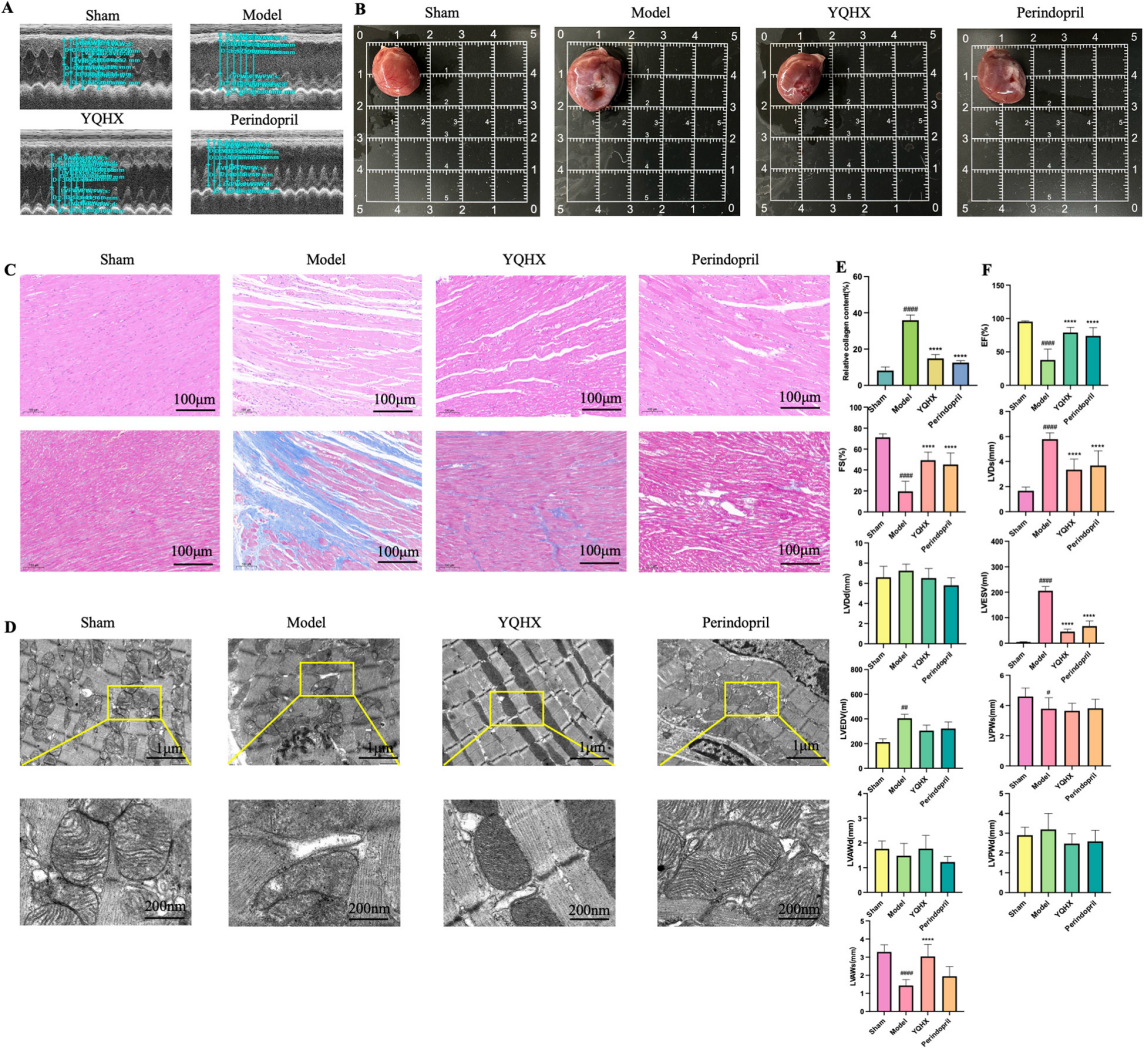
YQHX improved cardiac structure and dysfunction after MI **(A)** echocardiogram of rats in each group. **(B)** Macroscopic morphology of hearts tissue in each group. **(C)** HE and masson staining of heart tissues. **(D)** Ultrastructural observation of mitochondria, endoplasmic reticulum, and MAMs of hearts tissue under electron microscopy. **(E)** Myocardial fibrosis percentage in each group observed by masson staining. **(F)** Echocardiography measurements: EF, FS, LVDd, LVDs, LVEDV, LVESV, LVPWs, LVPWd, LVAWs, LVAWd. (*N* = 9).

Cardiac structure and function were evaluated through echocardiogram. 28 days after MI, the ejection fraction (EF) and fractional shortening (FS) were significantly reduced, while the left ventricular end-diastolic volume (LVEDV), end-systolic volume (LVESV), and left ventricular end-systolic diameter (LVDs) were significantly increased. However, both YQHX and perindopril did not show significant effects on end-diastolic diameter (LVDd) and LVEDV MI (Figures 1A,F). In S group, the cardiac appearance showed no obvious infarct area, with clear coronary vessels and appropriate heart size. While M group, a distinct pale infarct center was observed near the ligation site, accompanied by thinning and depression of the ventricular wall. Both YQHX and Perindopril group showed a smaller infarct area and clear and visible coronary vessels in the infarct border zone (Figure 1B). Similar, 7 days after MI, significant improvements in corresponding cardiac function and structure were also observed, suggesting that protective effects of both medicine on cardiac function and structure may be present during the subacute and chronic phases of MI.

HE staining is the most fundamental method for observing the pathological condition of myocardial tissue. After 28 days of MI, the myocardial cells in the S group were arranged neatly, and no significant inflammatory infiltration was observed in the extracellular matrix. Compared with the S group, the myocardial fibers in the M group were arranged chaotically, with structural ruptures and a decrease in the number of myocardial cells, accompanied by varying degrees of neutrophil infiltration. In contrast, the myocardial fibers in the Y and P groups were relatively neatly arranged, with regular cell morphology and significantly reduced inflammatory infiltration in the extracellular matrix (Figure 1C).

The degree of cardiac fibrosis was observed through masson staining. 28 days after MI, the myocardial fibers in S group mostly appeared red and were neatly arranged, with minimal collagen fibers. In contrast, the myocardial fibers in the M group were arranged chaotically, with widened fiber spaces and extensive blue stained collagen fibers in the infarcted area. Both YQHX and perindopril significantly improved the fibrosis of the myocardial tissue (Figures 1C,E). Mitochondria and endoplasmic reticulum are highly dynamic structures whose quality, morphology, localization, and composition undergo constant changes in response to the cellular needs. The regions where these two organelles exhibit physical and functional interactions are known as MAMs. Ultrastructural observations of myocardial tissue and cellular MAMs were conducted. S group exhibited neatly arranged myocardial fibers, with a small amount of scattered endoplasmic reticulum surrounding the mitochondria, and their connections were relatively loose. After MI, the myocardial fibers became disorganized, mitochondria were swollen with dilated endoplasmic reticulum surrounded, and the connections between mitochondria and endoplasmic reticulum became tighter. Following treatment with YQHX and perindopril, the myocardial fibers became more organized, mitochondrial morphology recovered, endoplasmic reticulum dilation decreased, and the connection distance between the two became relatively wider (Figure 1D). We also identified the same alteration trends of myocardial tissue, myocardial fibrosis, and mitochondrial-endoplasmic reticulum ultrastructure in the 7 days group.

### YQHX enhanced the vitality of H9c2 cells under glucose and oxygen deprivation conditions

**Figure 2:**
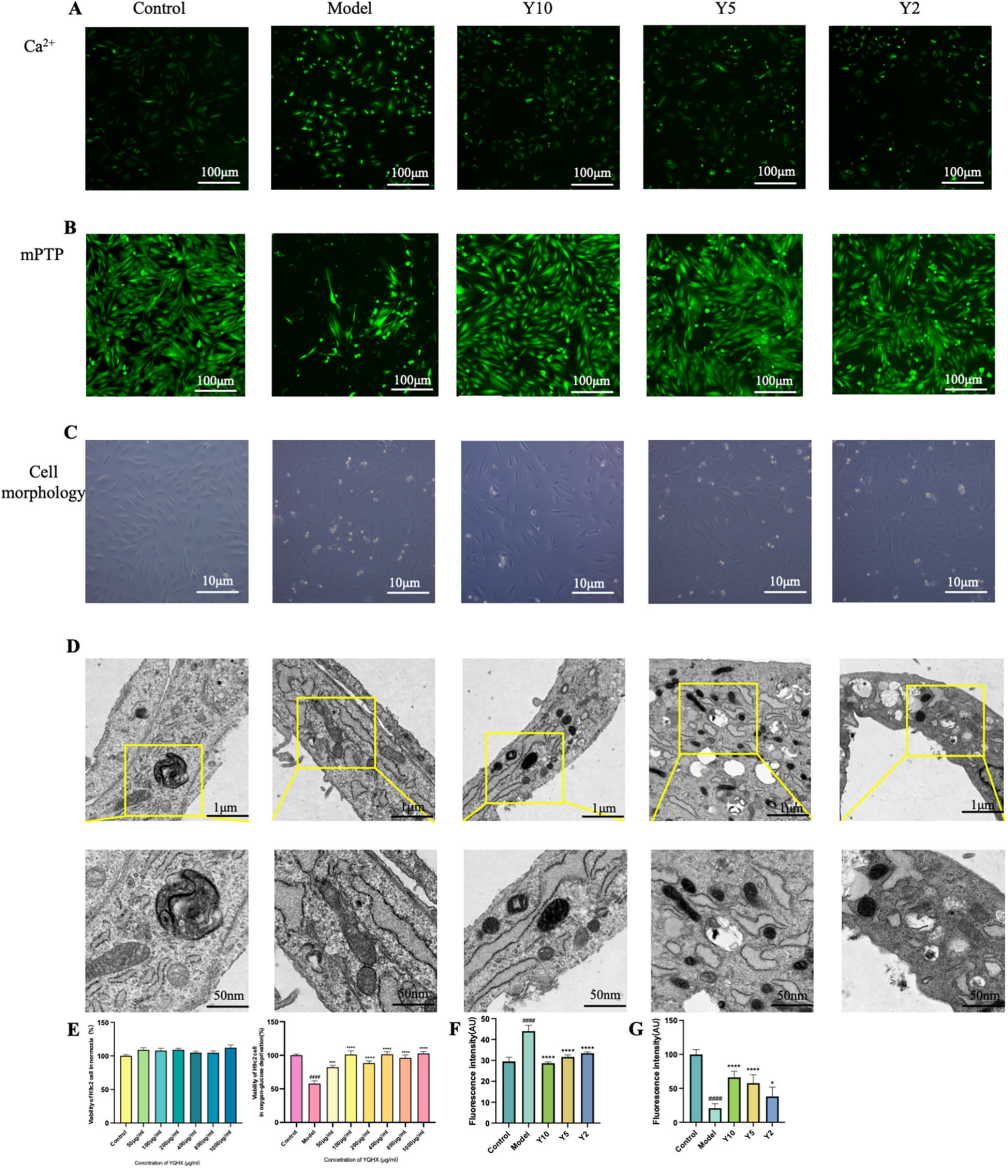
YQHX regulates the morphology of MAMs and Ca^2+^ flux in H9c2 cells **(A)** fluorescence microscopy observation of intracellular Ca^2+^ in each group of cells. **(B)** mPTP opening reflects the Ca^2+^ communication between ER and mitochondria. **(C)** Morphology of cells in each group via electron microscopy. **(D)** Ultrastructural observation of mitochondria, endoplasmic reticulum, and MAMs of H9c2 cells under electron microscopy. **(E)** CCK-8 assay to detect the effect of different concentrations of YQXH on cell viability under normoxic and hypoxic conditions. **(F)** Intracellular Ca^2+^ fluorescence intensity. **(G)** Calcein fluorescence intensity. (*N* = 3).

Drawing from our prior research (29), 100–400 μg/mL concentrations of YQHX significantly enhanced cell viability under hypoxic conditions for 24 h. This study expanded the concentration range and observed the effects of more concentrations on H9c2 cells. Under normoxic and glucose/oxygen-deprived conditions for 24 h, different concentrations of YQHX were used to intervene with H9c2 cardiomyocytes. Under normoxic conditions for 24 h, various concentrations of YQHX (ranging from 50 μg/mL to 1,000 μg/mL) had no significant impact on the vitality of H9c2 cells. Under glucose/oxygen-deprived conditions for 24 h, all concentrations of YQHX significantly improved the vitality of H9c2 cells. Considering the progressive concentration gradients set up in the experiment, we selected the concentrations of 1,000 μg/mL, 500 μg/mL, and 250 μg/mL for further research (Figures 2C,E). Like the heart tissues, in H9c2 cells, we found the same ultrastructural changes of MAMs. C group exhibited regularly shaped and full mitochondria, smooth and flat endoplasmic reticulum, with a certain gap between them. After the cells underwent glucose and oxygen deprivation, the mitochondria became swollen, with cristae fractured or even disappearing, and the endoplasmic reticulum became dilated and expanded, with a closer arrangement with the mitochondria. In the YQHX groups (Y10, Y5, Y2), the microstructure of MAMs showed significant improvement (Figure 2D).

### YQHX effectively regulates Ca^2+^ levels in H9c2 cells under glucose and oxygen deprivation conditions

Calcium, serving as a second messenger, occupies a pivotal role in the pathogenesis of cardiovascular diseases. In cardiomyocytes, calcium overload can induce cell apoptosis, leading to cell death and arrhythmias after MI (30). Through the study of Ca^2+^ levels in H9c2 cells, we found that glucose and oxygen deprivation conditions significantly increased intracellular Ca^2+^ concentration, leading to Ca^2+^ overload. YQHX significantly reduced the Ca^2+^ level in cardiomyocytes, indicating improvement function of Ca^2+^ overload in cardiomyocytes and reduction of cell damage (Figures 2A,F).

### YQHX reduced the excessive opening of mPTP in H9c2 cells induced by glucose and oxygen deprivation

The opening of mPTP can lead to dissipation of mitochondrial membrane potential, organelle swelling, and ultimately rupture. mPTP is defined as a large Ca^2+^-activated channel involved in mitochondrial damage and cell death (31). The research results showed that under normoxic conditions, mPTP was closed. Under glucose and oxygen deprivation conditions for 24 h, the opening of the mPTP in H9c2 cells was evident, leading to a significant decrease in fluorescence intensity. YQHX significantly improved the excessive opening of mPTP in H9c2 cells, thereby alleviating intracellular calcium overload (Figures 2B,G).

### YQHX improved the expression of MAMs-related and calcium regulation proteins

**Figure 3:**
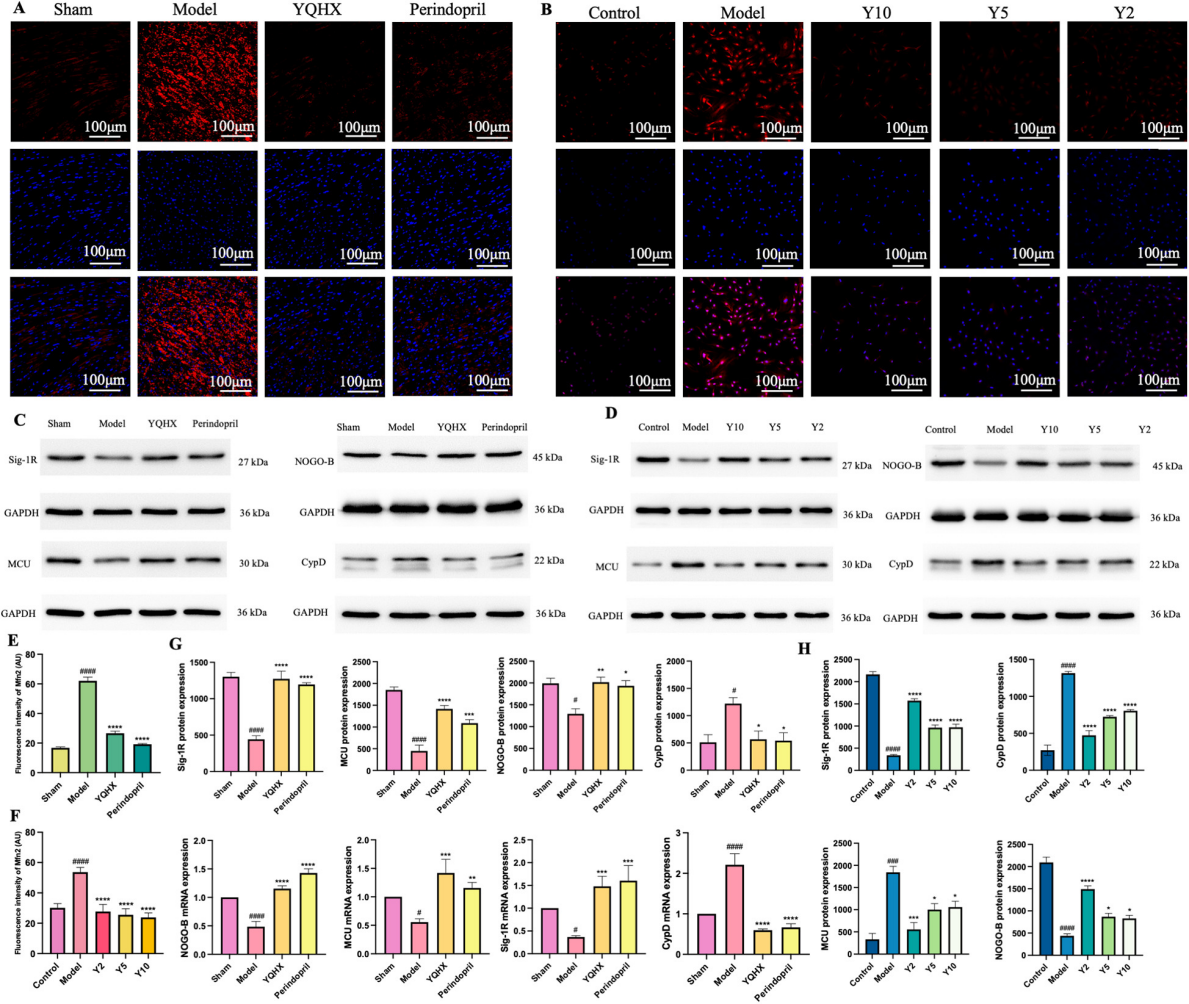
YQHX improved the expression of MAMs-related and calcium regulation proteins. **(A)** Mfn2 fluorescence staining of heart tissues in each group. **(B)** Mfn2 fluorescence staining of H9c2 cells in each group. **(C)** Western blot of MAMs-related calmodulin proteins (MCU, CypD, NOGO-B, Sig-1R) in heart tissues. (*N* = 3) **(D)** Western blot of MAMs-related calmodulin proteins (MCU, CypD, NOGO-B, Sig-1R) in H9c2 cells. (*N* = 3) **(E)** Mfn2 fluorescence intensity of heart tissues in each group. **(F)** Mfn2 fluorescence intensity of H9c2 cells in each group. (*N* = 3) **(G)** Expression of MAMs-related calmodulin proteins and genes (MCU, CypD, NOGO-B, Sig-1R) in heart tissues determined by Western blot and qPCR. (*N* = 9) **(H)** Expression of MAMs-related calmodulin proteins (MCU, CypD, NOGO-B, Sig-1R) in H9c2 cells determined by Western blot. (*N* = 3).

The mitochondrial fusion protein 2 (Mfn2) is not only targeted to mitochondria but also localized to the mitochondrial-associated endoplasmic reticulum membranes, regulating Ca^2+^ transport from the ER to mitochondria (32, 33). Both *in vivo* and *in vitro*, our experiments found that Mfn2 levels significantly increased under ischemic and glucose- and oxygen-deprivation conditions, while YQHX significantly improved the upregulation of Mfn2, thereby reducing the connections between MAMs (Figures 3A,B,E,F). Concurrently, *in vivo*, myocardial ischemia led to a significant increase in the calcium regulatory proteins cyclophilin D (CypD) and mitochondrial Ca^2+^ uniporter (MCU) on MAMs, while sigma-1 receptor (Sig-1R) and neurite outgrowth inhibitor B (NOGO-B) were significantly reduced. Both YQHX and perindopril reversed this state (Figures 3D,G). Similarly, *in vitro*, we observed the same results, indicating that YQHX may improve intracellular calcium overload by regulating relevant proteins on MAMs (Figures 3C,H).

### The impact of MKT-077 on the vitality of H9c2 cells

**Figure 4:**
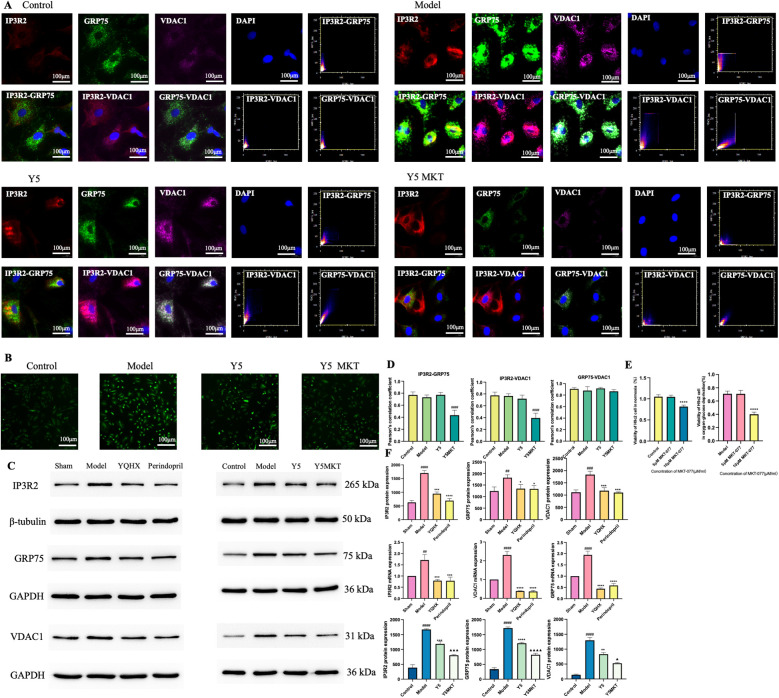
Effect of YQHX on the IP3R2-GRP75-VDAC1 complex and its related proteins. **(A)** Immunofluorescence colocalization images of the IP3R2-GRP75-VDAC1 complex and Pearson's correlation coefficient scatter plots. **(B)** Fluorescence microscopy observation of intracellular Ca^2+^ inhibited by MKT-077. (*N* = 3) **(C)** WB results of proteins related to the IP3R2-GRP75-VDAC1 complex in heart tissues and H9c2 cells. (*N* = 3) **(D)** Pearson's correlation coefficient of IP3R2-GRP75-VDAC1 complex colocalization. **(E)** Effects of different concentrations of MKT-077 on H9c2 cell viability under hypoxic and normoxic conditions. (*N* = 3) **(F)** Expression of the IP3R2-GRP75-VDAC1 complex in heart tissues and H9c2 cells detected by Western blot and qPCR. (*N* = 9) (*N* = 3).

GRP75, as a member of the HSP-70 family, can be inhibited by specific inhibitors of HSP70 (34). It has been previously shown that rhodacyanine dye MKT-077, a GRP75-specific inhibitor, binds to the nucleotide-binding domain of mortalin/GRP75, inducing tertiary structural changes that inactivate the ATPase activity of mortalin/GRP75 (35) preventing the calcium overload of nerve cell mitochondria in the oxygen and glucose deprivation model, leading to improved mitochondrial structure and function (36, 37). Notably, the GRP75 protein within the IP3R2-GRP75-VDAC1 complex belongs to the HSP-70 family and can also be inhibited by MKT-077. Given that the research application of MKT-077 in H9c2 cells is still limited, we first conducted concentration screenings using 5 μM and 10 μM of MKT-077 based on previous experimental studies (38, 39). Under both normoxia and glucose- and oxygen-deprivation conditions for 24 h, 5 μM/mL MKT-077 had no significant impact on the vitality of H9c2 cardiomyocytes. However, the vitality after intervention with 10 μM/mL MKT-077 was significantly lower compared to C and 5 μM/mL group, indicating that 10 μM/mL MKT-077 exhibits a certain degree of cytotoxicity towards H9c2 cardiomyocytes. Therefore, a concentration of 5 μM/mL was selected for the subsequent experiments (Figure 4E).

### MKT-077 reduced the Ca^2+^ levels in H9c2 cells

Compared with C group, the intracellular Ca^2+^ level in M group was significantly elevated. However, compared with M group, the intracellular Ca^2+^ levels in both Y5MKT and Y5 group were significantly reduced. This suggests that after GRP75 is blocked, the connection of the IP3R2-GRP75-VDAC1 complex is weakened, leading to a decrease in Ca^2+^ flow on the MAMs (Figure 4B).

### MKT-077 reduced the co-localization of IP3R2-GRP75-VDAC1 complex

The results of co-immunoprecipitations indicates that immunoprecipitation of both IP3R2 and VDAC leads to the co-precipitation of GRP75, and immunoprecipitation of GRP75 results in the co-precipitation of VDAC and IP3R2, suggesting that GRP75 plays a central role in the formation of the complex (40). Co-localization of IP3R2, GRP75, and VDAC1 was observed in C, M, and Y5 groups. However, compared to the first three groups, the co-localization of IP3R2 and GRP75, as well as IP3R2 and VDAC1, was significantly reduced in the Y5MKT group. (Figures 4A,D).

### YQHX improved the protein and gene expression of IP3R2-GRP75-VDAC1 complex

*in vivo*, the gene and protein expressions of IP3R2, GRP75, and VDAC1 were significantly upregulated 7 and 28 days after MI, while both YQHX and perindopril reversed this upregulation. Similarly, *in vitro*, YQHX significantly downregulated the high expressions of IP3R2, GRP75, and VDAC1 in H9c2 cells under glucose- and oxygen-deprivation conditions, and the decrease was even more significant in the Y5MKT group compared to the Y5 group, further confirming the blocking effect of MKT-077 on the complex (Figures 4C,F).

## Discussion

Myocardial infarction is the leading cause of death worldwide (41). Although the overall mortality rate related to MI has decreased globally, the mortality and incidence of heart failure associated with MI remain high. The American Heart Association estimates that the overall prevalence of acute MI is 3% (42). As a second messenger, Ca^2+^ plays an important role in various types of cells and is involved in various cardiovascular diseases such as hypertension, arrhythmias, and MI (43, 44). Calcium homeostasis imbalance is the main cause of cardiomyocyte death (45). Calcium homeostasis imbalance is not only an important cause of cardiomyocyte death but also a crucial factor leading to arrhythmias, myocardial hypertrophy, and heart failure. Calcium overload can induce apoptosis of cardiomyocytes, leading to MI (46, 47).

The formula of YQHX, an empirical prescription derived from the qi and blood theory in TCM is utilized in the clinical treatment of myocardial ischemia, which comprises Astragalus membranaceus, Angelica sinensis, Panax ginseng, Notoginseng, and Ligusticum wallichii. By clinically using for the treatment of MI, YQHX effectively improve patients' cardiac function and clinical symptoms (48).

Perindopril, used as a positive control drug is an angiotensin-converting enzyme inhibitor (ACEI) and a commonly used drug for improving clinical symptoms after MI (49). Perindopril has beneficial effects on hypertension, myocardial ischemia, atherosclerosis, as well as the structure and function of the cardiovascular system (50). As an ACEI, perindopril may not directly regulate myocardial calcium ions. However, it can bind to angiotensin receptor 1, activate guanine nucleotide-binding regulatory proteins, and subsequently trigger phospholipase C on the cell membrane to hydrolyze phosphatidylinositol bisphosphate into inositol trisphosphate (IP3) and diacylglycerol. IP3 binds to IP3 receptors on the endoplasmic reticulum, promoting Ca^2+^ release from the endoplasmic reticulum (51). In AngⅡ-induced H9c2 cells, this pathway can also regulate the opening of mitochondrial permeability transition pores (52).

In this experiment, we replicated a rat model of MI via LAD ligation to explore the protective effects of YQHX on ischemic myocardium. The findings revealed that both YQHX and perindopril alleviated the cardiac structural changes and dysfunction induced by MI. Based on the results of EF, FS, LVEDV, LVESV, LVDd, LVDs, it was suggested that both YQHX and perindopril exerted more significant effects during the subacute and chronic phase of MI. The proteins located on MAMs that are involved in Ca^2+^ regulation mainly include: Mfn2, MCU, CypD, NOGO-B, Sig-1R, etc al. Mfn2, as a component of the mitochondrial network remodeling mechanism, is targeted and localized to mitochondria, participating in the formation of endoplasmic reticulum-mitochondria contacts (53) and regulating the transport of Ca^2+^ between the two organelles (33). Our previous research results on Mfn2 showed significantly reduced at gene and protein level after MI, YQHX and perindopril could significantly increase these expression (23). Interestingly, in both *in vivo* and *in vitro* found that after myocardial cells experienced ischemia, glucose deprivation and hypoxia, the expression of Mfn2 was significantly upregulated, leading to an increase in mitochondrial fusion proteins and subsequent myocardial and mitochondrial damage. However, YQHX downregulated the expression of Mfn2, thereby facilitating the recovery of damaged myocardium and mitochondria. These findings are consistent with the ultrastructural of MAMs, further indicating that YQHX can repair the ultrastructural damage of myocardial cells by reducing the expression of mitochondrial fusion proteins. The contradiction in these conclusions can be explained by the fact that there is still some controversy surrounding the role of Mfn2 in Ca^2+^ regulation and cell death as part of the mitochondrial-endoplasmic reticulum tether. Filadi et al. (54) demonstrated that silencing of Mfn2 increased the close apposition between the two intracellular structures and enhanced the efficiency of Ca^2+^ transfer from the endoplasmic reticulum to mitochondria. Naon et al. (55) observed that the ablation of Mfn2 led to an increase in the distance between organelles, resulting in a reduced capacity of mitochondria to sequester Ca^2+^ released from the endoplasmic reticulum. Similarly, Hall AR et al. (56) demonstrated that acute elimination of Mfn2 in the heart confers resistance to acute MI. The disruption of the interaction between mitochondria and the sarcoplasmic reticulum can reduce mitochondrial Ca^2+^ overload.

MCU and CypD, as intracellular calcium regulatory proteins, play a crucial role in maintaining calcium homeostasis in cells. The mitochondrial Ca^2+^ uptake is regulated by MCU within the mitochondrial membrane (57). Target knockout of MCU in the heart results in a decrease in infarct size and an enhancement in cardiac function following ligation of the LAD (58). Ting Liu et al. (59) found that overexpressed MCU during cardiac decompensation may reverse the course of heart failure, improve the contractile response of cardiomyocytes, and suppress HF-related arrhythmias by enhancing mitochondrial Ca^2+^ accumulation, reducing mitochondrial oxidative stress, and mitigating SR Ca^2+^ leakage. MCU and mPTP jointly regulate Ca^2+^ homeostasis. Elevated levels of mitochondrial calcium can lead to cell death by activating mPTP (60). Mei-ling A. Joiner et al. (61) found that calmodulin-dependent protein kinase II promotes the opening of mPTP and cardiomyocyte death by increasing MCU current. CypD, as a cyclophilin in the mitochondrial matrix, is also closely related to the opening of mPTP and plays a role in mitochondrial calcium regulation and cell apoptosis (62). Ablation or inhibition of CypD enhances the resistance of mitochondria to Ca^2+^-induced cell swelling (63). In cardiovascular diseases, inhibition of CypD blocked the activation of mPTP in isolated mitochondria, thereby reducing cell death during ischemia-reperfusion injury in the heart (64).This study investigated the expression of MCU and CypD *in vivo* and *in vitro*, respectively. The results showed that cardiomyocytes mPTP opens significantly with the influx of Ca^2+^ under conditions of glucose deprivation and hypoxia. The protective effect of YQHX on cardiomyocytes may stem from its upregulation of MCU, which regulates the opening of mPTP and maintains Ca^2+^ homeostasis. Additionally, YQHX may also block the activation of mPTP by inhibiting CypD, relieving calcium overload in cells, and exerting a protective effect on the myocardium. These two mechanisms may be potential targets for the treatment of MI with YQHX.

NOGO-B is also a calcium-binding protein located on MAMs. Studies have proven that NOGO-B is expressed in the heart and regulated cardiovascular diseases such as cardiac myocardial ischemia, atherosclerosis, and hypertrophy (65, 66). Increased expression of NOGO-B can induce the expansion between mitochondria and the endoplasmic reticulum, reduce intracellular Ca^2+^ levels, and inhibit mitochondria-mediated apoptosis (67). Hypoxia induced upregulation of NOGO-B expression and potentially disrupt the MAMs unit by altering the structure of the endoplasmic reticulum. In contrast, the absence of NOGO-B maintained the structural integrity of MAMs unit, resulting in reduced mitochondrial Ca^2+^ content (68). YQHX reversed the low expression of NOGO-B in ischemic and hypoxic cardiomyocytes, maintains the structural integrity of MAMs, and alleviates cardiomyocyte death after MI by improving Ca^2+^ flux on MAMs. This suggests that NOGO-B may play different roles in maintaining MAMs structure in different types of cells.

Sig-1R is a molecular chaperone located on the endoplasmic reticulum membrane and is positioned at MAMs (69). It can produce a cardioprotective effect by improving myocardial energy metabolism (70). Sig-1R plays a role in stabilizing and activating IP3R in maintaining Ca^2+^ regulation. Through its interaction with IP3R, Sig-1R ensures appropriately Ca^2+^ flow from the endoplasmic reticulum into mitochondria, thereby maintaining normal mitochondrial function and Ca^2+^ homeostasis (71). This study found that YQHX improved the low expression of Sig-1R after MI. Combined with the results of MAMs microstructure and Ca^2+^ levels, it can be inferred that the mechanism of YQHX in treating MI may be through regulating Ca^2+^ flux on MAMs via Sig-1R, improving the structure of MAMs, and subsequently improving cardiac function, exerting a cardioprotective effect. Additionally, further evidence that YQHX can reduce cardiomyocyte death by regulating Ca^2+^ flux on MAMs and alleviating intracellular calcium overload *in vitro* has been proved.

IP3Rs, GRP75, and VDAC1 serve as typical components of the Ca^2+^ transfer unit in MAMs, providing fuel for the mitochondrial low-affinity Ca^2+^ uptake system. Studies have found that VDAC1 is significantly overexpressed in patients after MI (72). Zhao et al. discovered that during the development of cardiac hypertrophy, the MAMs channel proteins VDAC1 and GRP75 are significantly upregulated (73). GRP75 can connect IP3Rs and VDAC1 through its cytosolic portion, forming an IP3Rs/GRP75/VDAC1 channel complex that mediates mitochondrial Ca^2+^ uptake (74). Accumulating evidence has demonstrated that all three isoforms of IP3R localize to endoplasmic reticulum-mitochondria contact sites (ERMCS), where they orchestrate Ca^2+^ signaling. Among these, IP3R2 serves as the predominant isoform in cardiomyocytes (75). IP3R2 is the most effective in mediating Ca^2+^ transfer to mitochondria. Therefore, we temporarily choose IP3R2 as the research subject (76, 77). The three proteins of the IP3R2-GRP75-VDAC1 complex play a role in regulating Ca^2+^ on MAMs respectively. Ying Qi et al. (78) found that the sarcoplasmic reticulum Ca^2+^ released by IP3R2 is crucial for the Ca^2+^ microdomains and mitochondrial Ca^2+^ uptake in cardiomyocytes. An increase in GRP75 expression accelerates the transfer of Ca^2+^ from the endoplasmic reticulum to mitochondria, leading to Ca^2+^ overload (79). VDAC1 regulates the flow of Ca^2+^ through MAMs and GRP75, modulating mitochondrial Ca^2+^ homeostasis as well as the interaction between Ca^2+^ and IP3 receptors in the endoplasmic reticulum (33). Studies have found that VDAC1 expression increases after MI (7). Under conditions of cardiac ischemia/reperfusion, upregulated VDAC1 expression can increase myocardial cell damage (80). Additionally, the expression level of VDAC1 significantly increases in H9c2 cells after oxidative stress injury (81). Co-immunoprecipitation results showed that immunoprecipitation of both IP3Rs and VDAC led to the co-precipitation of GRP75, and immunoprecipitation of GRP75 resulted in the co-precipitation of VDAC and IP3R. This indicates that GRP75 plays a central role in the formation of protein complexes with VDAC and IP3R (40). Through immunofluorescence detection, our study initially observed the co-localization of IP3R2-GRP75-VDAC1. However, MKT-077 significantly reduced the co-localization between IP3R2 and GRP75, as well as between IP3R2 and VDAC1, indicating that the GRP75 blocker-MKT-077 disrupted the connection between IP3R2 and VDAC1 to a certain extent. This conclusion is further supported by the reduced Ca^2+^ levels observed. When the bridging role of GRP75 is blocked, the flow of Ca^2+^ on MAMs is interrupted, which alleviates intracellular calcium overload to a certain extent. Furthermore, YQHX appears to enhance the interruption of Ca^2+^ flow within the complex, suggesting that its ability to improve calcium overload in cardiomyocytes may be mediated through the IP3R2-GRP75-VDAC1 complex. YQHX exerts a cardioprotective function by improving the structure of mitochondria, endoplasmic reticulum, and MAMs in ischemic myocardium and H9c2 cells deprived of glucose and oxygen. It regulates the expression of MAMs-related and calcium-regulating proteins such as MCU, CypD, NOGO-B, and Sig-1R, and decreases the expression of IP3R2, GRP75, and VDAC1. Additionally, it modulates the opening state of mPTP and alleviates intracellular calcium overload through the IP3R2-GRP75-VDAC1 complex. Therefore, MAMs and the IP3R2-GRP75-VDAC1 complex represent potential targets and significant pathways for YQHX to regulate calcium overload and exert cardioprotective effects after MI.

## Data Availability

The datasets presented in this study can be found in online repositories. The names of the repository/repositories and accession number(s) can be found in the article/Supplementary Material.
